# The effect of obesity on revision rate in unicompartmental knee arthroplasty: a systematic review and meta-analysis

**DOI:** 10.1007/s00167-020-06297-7

**Published:** 2020-10-16

**Authors:** Omar Musbahi, Thomas W. Hamilton, Adam J. Crellin, Stephen J. Mellon, Benjamin Kendrick, David W. Murray

**Affiliations:** 1 Nuffield Department of Orthopaedics, Rheumatology and Musculoskeletal Sciences, Botnar Research Centre, University of Oxford, Windmill Road, Oxford, OX3 7LD UK; 2 Medical Sciences Division, John Radcliffe Hospital, Headley Way, Oxford, OX3 9DU UK

**Keywords:** Unicompartmental knee arthroplasty, Unicondylar knee arthroplasty, Obesity, Body mass index, Reoperation

## Abstract

The number of patients with knee osteoarthritis, the proportion that is obese and the number undergoing unicompartmental knee arthroplasty (UKA) are all increasing. The primary aim of this systematic review was to determine the effects of obesity on outcomes in UKA. A systematic review was performed using PRISMA guidelines and the primary outcome was revision rate per 100 observed component years, with a BMI of ≥ 30 used to define obesity. The MINORS criteria and OCEBM criteria were used to assess risk of bias and level of evidence, respectively. 9 studies were included in the analysis. In total there were 4621 knees that underwent UKA. The mean age in included studies was reported to be 63 years (mean range 59.5–72 years old)) and range of follow up was 2–18 years. Four studies were OCEBM level 2b and the average MINORS score was 13. The mean revision rate in obese patients (BMI > 30) was 0.33% pa (95% CI − 3.16 to 2.5) higher than in non-obese patients, however this was not statistically significant (*p* = 0.82). This meta-analysis concludes that there is no significant difference in outcomes between obese and non-obese patients undergoing UKA. There is currently no evidence that obesity should be considered a definite contraindication to UKA. Further studies are needed to increase the numbers in meta-analysis to explore activity levels, surgeon’s operative data, implant design and perioperative complications and revision in more depth.

**Level of evidence** Level III.

## Introduction

The incidence and prevalence of obesity in the Western World is increasing [3]. According to the World Health Organisation (WHO), the prevalence of obesity has nearly doubled worldwide over the past three and half decades, particularly in developing countries [3]. Obesity is considered a major risk factor for osteoarthritis, thus the implications of this increase directly correlate to the increase in osteoarthritis (OA) and in particular knee OA [41]. Studies have shown that with every 5 kg of weight gain the risk of knee OA increases by 36% [25]. Whilst initial management of knee OA is non-operative, some patients require arthroplasty as the disease progresses and conservative methods become ineffective. Hence with the increase in knee OA, there has been research into the indications and effects of arthroplasty in this group of patients, in particular the use of unicompartmental knee arthroplasty (UKA).

Big data studies have suggested that high UKA revision is due to a caseload effect. Hence low volume surgeons and low centres have an associated higher revision rate [4, 16]. There is an assumption that UKA has a higher revision rate than total knee arthroplasty (TKA) primarily because it is easier to revise. In addition, it has other advantages, such as a more rapid recovery, fewer complications and better function. Therefore, there may be an argument that UKA may be the optimal choice for obese patients, particularly if they are young. Proponents of UKA believe that UKA can potentially be used in up to 50% of patients needing knee replacements, with the proportion being higher in younger patients [20].

However, since Kozinn and Scott highlighted in their seminal paper in 1989 that UKA is not ideal with a body weight of > 82 kg [23], most surgeons have avoided using UKA in obese patients. Despite this, some surgeons have used unicompartmental knee arthroplasty (UKA) in this situation and have challenged the view that obesity should be considered to be a contraindication [15, 35]. For example, Caivagnac showed that obesity had no adverse outcomes in UKA with a 92% 10-year survival rate [11]. Similarly, Murray et al. in a prospective study of 2438 Oxford UKA found that increasing BMI was not associated with an increasing failure rate [30]. In contrast, Kandil et al. found the overall short-term revision rate in obese patients undergoing UKA was twice as high as the revision rate in non-obese patients [18].

There is currently no published systematic review and meta-analysis evaluating the effects of obesity on UKA. Current studies investigating the effect of obesity on the outcome of UKA have been limited by short term follow-ups [24, 31], few revisions [11, 42] and small cohorts [6, 31, 38, 43]. Hence the aim of this systematic review and meta-analysis was to identify the influence of obesity, defined as a BMI as ≥ 30, on outcomes of UKA. In addition, it aims to determine whether increased BMI represents a risk factor for failure and if it does, what BMI is associated with worse outcomes and what the mechanisms of failure are. Thus, the null hypothesis is that there is no difference in revision rate for obese patients undergoing a UKA compared to non-obese patients. This study should inform surgical decision-making in UKA patient selection to improve outcomes, answer an important research question and add to the growing knowledge of the optimal management of knee OA in obese patients.

## Patients and methods

### Search methods

PubMed, Ovid and Web of Science were searched for clinical studies that reported the influence of BMI on outcomes following UKA for osteoarthritis between 1980 to June 2020. Obesity was defined as a BMI ≥ 30. The search terms were set with the aid of a trained librarian (Appendices). Titles and abstracts were screened for relevance using the Preferred Reporting in Systematic Reviews and Meta-analysis (PRISMA) guidelines [29].

### Outcomes of interest

The primary research question was to determine whether the outcome of UKA was influenced by BMI. The primary outcome of interest was revision rate due to any cause. Initial comparison was between obese patients (BMI ≥ 30) and non-obese patients (BMI < 30). Subgroup analysis was undertaken to compare patients with a BMI ≥ 35 and BMI < 35 undergoing UKA to further evaluate the ideal BMI cut off.

Subgroup analysis was also performed by failure mechanism, specifically assessing revision for infection, aseptic loosening and unexplained pain. These three secondary outcomes were chosen, as they are main determinants of failure in UKA [28, 39]. The influence of prosthesis design, fixed vs. mobile bearing, will also be assessed. Postoperative Oxford Knee Score (OKS) and Knee Society Score (KSS) will also be evaluated comparing the two BMI groups.

### Selection criteria

Comparative studies that compared revision rates for obese (BMI ≥ 30) and non-obese patients (< 30) were included. Studies that compared revision rates for patients with BMI ≥ 35 and BMI < 35 were also included.

All types of UKA prosthesis designs (fixed bearing, mobile bearing) were included. Only studies with a minimum 1 year follow-up period were included. Any articles where the data were not extractable was excluded. The remaining inclusion and exclusion criteria are highlighted in Table 1.

**Table 1:** Table of inclusion and exclusion criteria used in the systematic review

Inclusion Criteria
Comparative studies in which the outcomes were reported according to BMI Observational studies and randomised controlled trials Minimum 1 year follow up period All types of unicompartmental knee prosthesis designs Full text available Data must be extractabl
Exclusion Criteria
Review articles, expert opinions, surgical techniques and abstracts from scientific meetings (data not available) Studies not in English language

### Data extraction and analysis

Two independent reviewers (OM and AC) performed the screening process in line with PRISMA guidelines. Following database screening, the remaining articles were analysed. Duplicates were removed. Demographic data extracted from each study included mean age, follow up period, number of knees and number of patients.

### Level of evidence

The methodology of the studies was assessed using the list of criteria as recommended by the Cochrane Collaboration and an Oxford Centre for Evidence-Based Medicine (OCEBM) level of evidence (LoE) was assigned to each study [32]. A LoE of 1 is the highest level of evidence and assigned to a high-quality randomised control trial or meta-analysis.

### Risk of bias

Risk of Bias was assessed using the Methodological Index for Non-Randomised Studies (MINORS) criteria [37]. The MINORS criteria are a 12-item checklist that assigns a score on various parameters to assign an overall risk of bias score out of 24 (low level risk of bias). MINORS assessment was performed to ensure there was a descriptive summary of source of bias in each study. No study was excluded based on this assessment.

### Statistical analysis

The data from the included studies were pooled and analysed using REVMAN (Cochrane Collaboration, Copenhagen) review manager statistical software and R (R Core Team, Vienna) statistical software programme.

The total revision rate per 100 observed component years was calculated by multiplying the number of cases by the mean follow-up for each study. The total number of revisions was then divided by the total observed component years and multiplied by 100. The 95% confidence intervals were calculated using the Clopper Pearson exact method [10].

The weighted odds ratio and 95% confidence interval (CI) was calculated for dichotomous variables. The weighted means difference was calculated for continuous variables. The heterogeneity of the included studies was calculated using the *I*^2^ statistic to describe the percentage of variation across studies that is due to heterogeneity rather than chance [17]. A random effects model was used. A chi-square *p* value < 0.1 was suggestive of statistical heterogeneity.

## Results

### Study selection

**Fig. 1:**
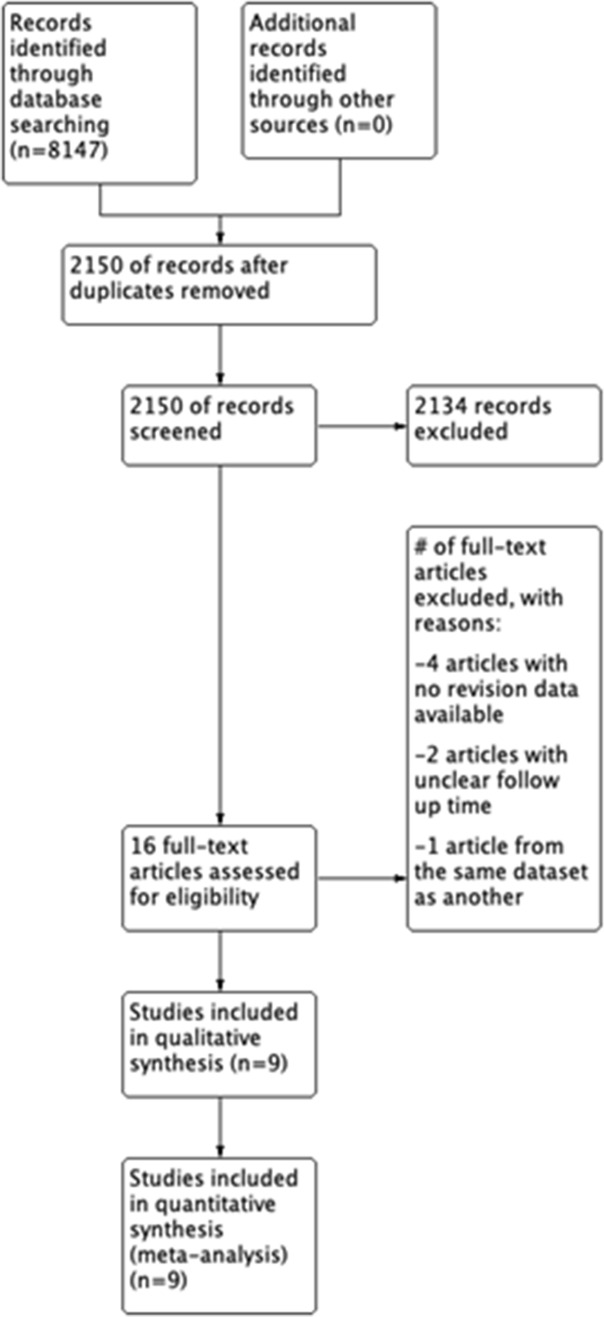
PRISMA flow diagram of record identification, screening and selection in meta-analysis

The PRISMA flow diagram showing selection of studies is illustrated in Fig. 1. Nine studies were included in the analysis having met the inclusion criteria. Four (44%) studies were prospective and five were retrospective. No studies were randomised. Eight (89%) studies could be used for a comparison of patients undergoing UKA with BMI above and below 30 and four (44%) studies could be used for comparison of patients with BMI above and below 35. In total, there were 4621 knees with a mean age of 63 years (range) that underwent UKA. Table 2 summarises the baseline characteristics and demographics of the studies.

**Table 2:** Study characteristic, demographics and LoE of studies meeting inclusion criteria

Study	Year	Type of Study	Type of Prosthesis (Fixed bearing/mobile bearing)	No. of Patients	No. of Knees	mean Age, range (years)	BMI categories	Follow-up period (months)	Secondary outcomes of Interest	Level of Evidence (LoE)
Seyler [36]	2009	Cohort	Fixed bearing	68	80	72(44–91)	< 30, > 30	60 months (24–128)	–	2b
Bonutti [8]	2011	Retrospective	Fixed bearing	67	80	66.5(45–81)	< 35, > 35	36 months (24 to 84)	–	3b
Plate [33]	2015	Retrospective	Fixed bearing	672	746	64	< 18.5, 18.5–25, 25–30, 30–35, 35–40, 40–45, > 45	33.6 months (24 +)	–	3b
Zengerink [45]	2015	Retrospective	Fixed bearing	130	141	60.5	< 30, > 30	60 months (24–144)	Unexplained pain	3b
Venkatesh [40]	2016	Cohort	Fixed bearing	148	175	61.7	< 30, > 30	67.2 months (24–120)	Unexplained Pain, aseptic loosening, KSS	2b
Woo [42]	2017	Retrospective	Fixed bearing	673	673	62	< 25, 25–30, 30–35, > 35	64 months (30–102)	Unexplained pain, aseptic loosening	3b
Murray [30]	2013	Cohort	Mobile bearing	–	2438	64	< 25, 25–30, 30–35, > 35, 35–40, 40–45, > 45	55.2 months (24–144)	Unexplained pain, infection, aseptic loosening, OKS, KSS	2b
Xu [44]	2019	Cohort	Fixed bearing	184	184	59.5	> 30, < 30	Minimum 120 months	Aseptic loosening, infection, KSS, OKS	2b
Polat [34]	2019	Retrospective	Mobile bearing	104	104	60.2	> 30, < 30	46 months (31.4– 60.6)	Aseptic loosening, infection	3b

### Level of evidence

Five (56%) studies had Level 3b evidence and the remainder were Level 2b based on the OCEBM criteria (Table 2). This suggests the results of the meta-analysis may assist in forming basis of recommendations for revision rate, aseptic loosening, infection and aseptic loosening outcomes in the obese population.

### Risk of bias

The mean MINORS score was 13 (range 11–20) as shown on Table 3. All the studies had clearly stated aims, adequate statistical analysis and appropriate endpoints. However, most of the studies were not prospective and did not state the baseline equivalence of groups. Furthermore, almost all the studies did not adjust for possible confounding factors in obese patients undergoing UKA; however, due to the nature of the study research question it is likely that every study will have been expected to score low on this metric. Hence, the true effect of obesity on UKA outcomes should be interpreted in context.

**Table 3:** Risk of Bias assessment using the MINORS criteria for each study

Study	Clearly stated aim	Inclusion of consecutive patients	Prospective collection of data	Endpoint appropriate to the aim of the study	Unbiased assessment of the study endpoint	Follow up period appropriate to the aim of the study	Loss of follow up less than 5%	Prospective calculation of the study size	An adequate control group	Contemporary group	Baseline equivalence of groups	Adequate statistical analysis	Total
Seyler [36]	2	1	2	2	0	2	0	0	2	1	0	2	14
Bonutti [8]	2	0	0	2	0	2	0	2	2	1	0	2	13
Plate [33]	2	1	0	2	0	2	2	0	2	1	0	2	12
Zengerink [45]	2	0	0	2	0	2	2	0	2	1	0	2	13
Venkatesh [40]	2	0	0	2	0	2	1	0	2	1	0	2	12
Woo [42]	2	0	0	2	0	0	0	0	2	1	0	2	11
Murray [30]	2	2	2	2	0	2	2	2	2	2	0	2	20
Xu [44]	2	0	0	2	0	2	0	0	2	1	0	2	11
Polat [34]	2	1	0	2	0	2	0	0	2	1	0	2	12

### Meta-analysis

**Fig. 2:**
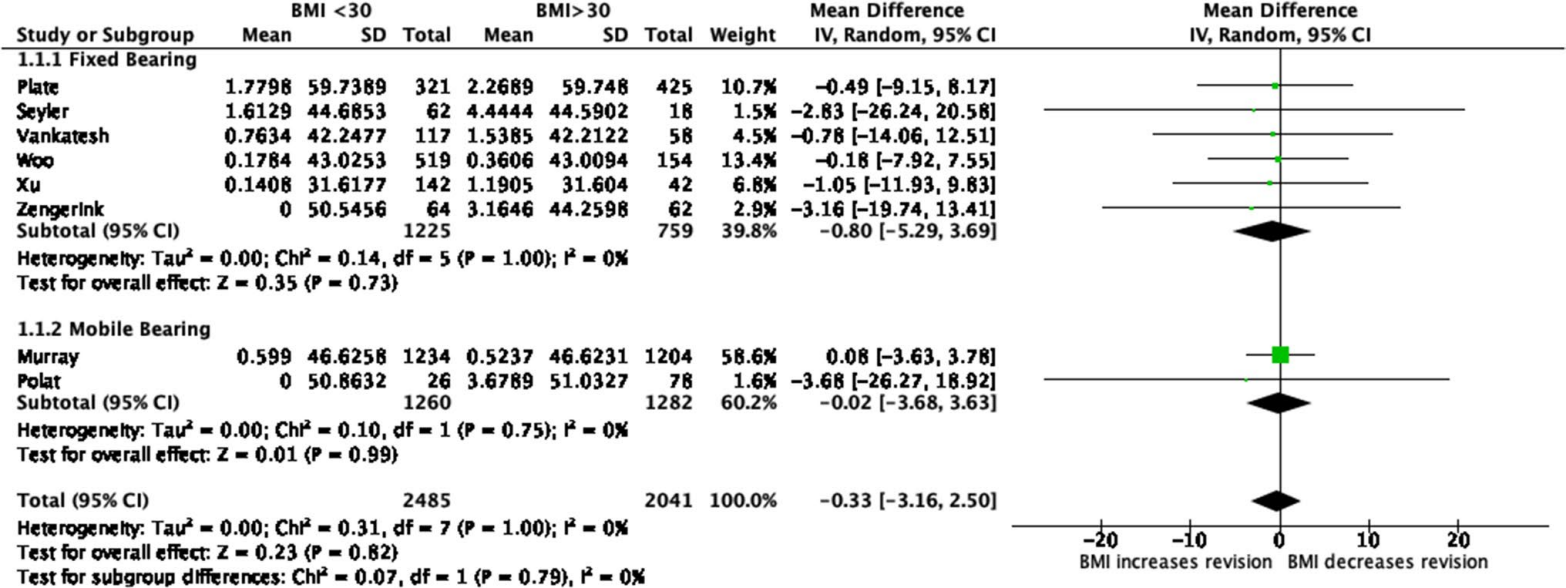
Forest plot of overall revision rate after UKA comparing BMI ≥ 30 and BMI < 30

Table 4 highlights the revision rate and observed component years for each study. The revision rate of UKA in studies of patients with BMI ≥ 30 was 0.33% pa higher than in patients with a BMI < 30. The difference was however not statistically significant (*p* = 0.82) and was small as compared to the 95% confidence interval (CI), which was from − 2.5% pa to 3.16% pa. (Fig. 2).

**Table 4:** Study results showing observed component years and all cause annual revision rate comparing obese (BMI > 30) and non-obese (BMI < 30) patients

Study	Year	Observed component years		Annual revision rate (%)	
		BMI < 30	BMI > 30	BMI < 30	BMI > 30
Seyler [36]	2009	310	90	1.612	4.444
Bonutti [8]	2015	899	1190	1.779	2.269
Plate [33]	2015	250	316	0	3.164
Zengerink [45]	2016	655	325	0.763	1.538
Venkatesh [40]	2017	2803	832	0.178	0.361
Woo [42]	2013	5676	5538	0.599	0.524
Murray [30]	2019	1420	420	0.141	1.190
Xu [44]	2019	100	299	0	3.68

**Fig. 3:**
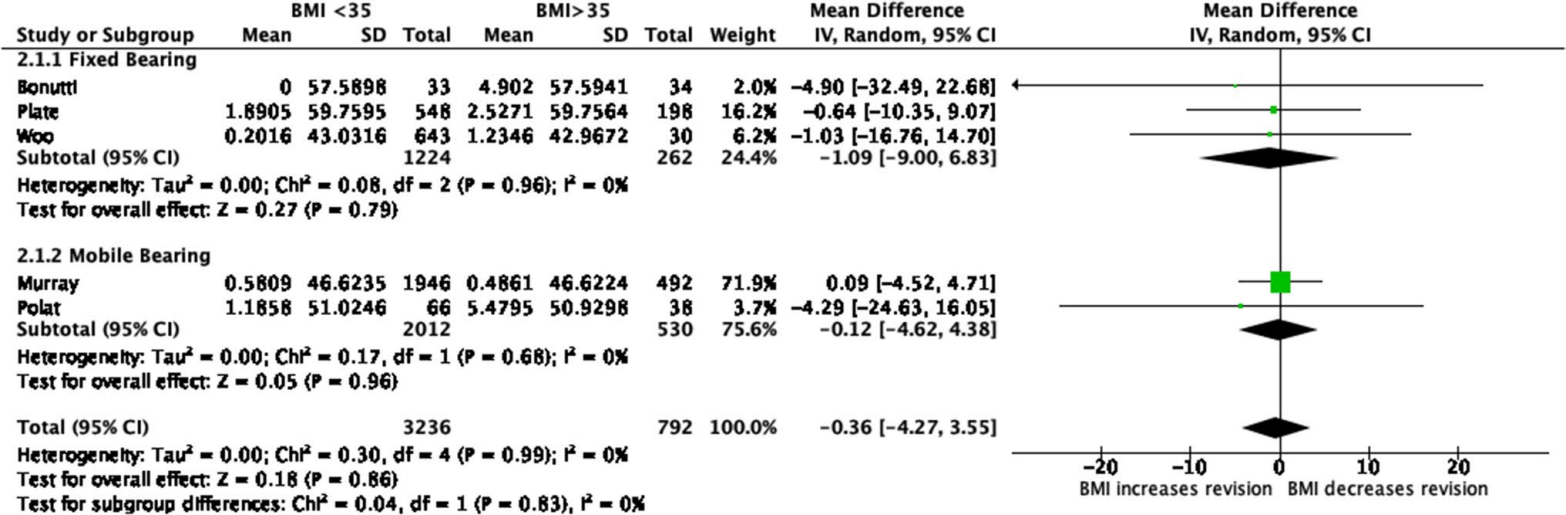
Forest plot of overall revision rate after UKA comparing BMI ≥ 35 and BMI < 35

As shown in Fig. 3, the revision rate of UKA in studies of patients with BMI ≥ 35 was 0.36% pa higher than in patients with a BMI < 30. The difference was; however, not statistically significant (*p* = 0.83) and was small as compared to the 95% (CI), which was from − 3.55% pa to 4.27% pa. This was associated with a low level of heterogeneity (*I*^2^ = 0%).

Meta-analysis of subgroup analysis was performed based on the failure mechanism and implant design as shown on Table 5. From the subgroup meta-analysis (Table 5), there was no difference in the secondary outcomes except for unexplained pain and OKS outcome.

**Table 5:** Meta-analysis summary of primary outcomes for revision rate comparing both BMI’s and secondary outcomes using BMI cut off of 30

Outcome	Number of knees	Number of events	Heterogeneity, *I*^2^ (%)	*p* value	Odds ratio or mean difference (95% CI)
Overall revision rate (BMI > 30, BMI < 30)	4526	149	0	0.82	MD − 0.18 (95% CI − 2.36 to 2.01)
Overall revision rate (BMI > 35, BMI < 35)	4028	104	0	0.86	MD -0.36 (95% CI − 4.27 to 3.55)
Infection revision rate (BMI > 30, BMI < 30)	757	10	0	0.41	OR 1.73 (95% CI 0.47 to 6.31)
Aseptic loosening revision rate, BMI > 30, BMI < 30)	975	6	0	0.52	OR 1.56 (95% CI 0.41 to 5.99)
Unexplained pain revision rate (BMI > 30, BMI < 30)	974	13	0	0.04	OR 3.66 (95% CI 1.09 to 12.30)
Revision rate (mobile bearing, BMI > 30, BMI < 30)	2542	49	0	0.99	OR − 0.02(95% CI − 3.16 to 2.50)
Revision rate (fixed bearing, BMI > 30, BMI < 30)	1984	83	0	0.73	OR -0.80 (95% CI, − 5.29 to 3.69)
KSS (BMI > 30, BMI < 30)	359	–	45	0.32	MD 0.32 (95% CI − 2.2 to 2.84)
OKS (BMI > 30, BMI < 30)	1110	–	0	< 0.05	MD − 1.81 (95% CI − 2.75 to − 0.86)

### Prosthetic design

Fixed bearing prosthesis was not associated with an increased risk of revision in obese patients (means difference 0.80, 95% CI − 5.29 to 3.69) and neither was mobile bearing prosthesis (means difference 0.02, 95% CI − 3.16 to 2.50) (Table 5).

## Discussion

The study showed that there was no significant increase in the risk of revision of UKA implanted in obese patients. This was the finding whether the comparison was made between patients with a BMI above or below 30 and 35. Therefore obesity does not represent a clear risk factor for failure and should not be considered a definite contraindication for UKA. However, for patients with BMI above both 30 and 35 there was an increase in the mean revision rate (0.33% pa and 0.36% pa), which was substantially smaller than the very wide confidence intervals (− 2.5 to 3.16 and − 3.55 to 4.27). This equated to only a small difference of 3–4% in revision rate at 10 years. Furthermore, the risk of bias in the studies was appreciable (Table 3) and there was low heterogeneity in the meta-analysis. Therefore, obesity may be associated with an increased risk of revision so surgeons should continue to be cautious implanting UKA in obese patients, who should be warned that there might be an increased risk of revision.

Every patient that is treated with a UKA could have had a TKA instead. Therefore, when deciding whether to do a UKA in obese patients, it is sensible to consider not just the possible increased revision rate of UKA in the obese, but also the increased revision rate in TKA. A high-quality meta-analysis of 20 studies (15,276 patients) by Kerkhoff showed that the revision rate is higher in the obese (BMI > 30) population undergoing TKA (OR 1.79, 95% CI 1.15–2.78) [21]. Although our study used different methodology and had fewer patients, the increase in mean revision rate with obesity following UKA of about 20% was lower than the increase of about 80% that following TKA. Therefore, if there is a slight increase in revision rate following UKA with obesity this should not necessarily be considered a contraindication and might be an argument for obese patients to undergo a UKA rather than a TKR. Further analysis is warranted to compare outcomes of obese patients undergoing UKA and TKA.

Currently there is no data from registry studies that has looked at obesity outcomes in UKA. This may be due to several factors including caseload effect, threshold of revision and incomplete data. In particular the UKA registry data seem to have significant problems with missing BMI data and in a study by Liddle et al. using the National Joint Registry (NJR) data reporting 52.9% of patients out of 41,986 did not have a recorded BMI [26]. In contrast to UKA, there seems to be clear agreement between meta-analysis in the literature and registry data on TKA and obesity outcomes with greater evidence available regarding the higher revision rate in TKA [9, 12]. Erdem et al. from the Danish knee registry found that survival was affected in patients over the age of 70 years weighing < 60 kg and > 80 kg but weight was not found to affect risk of revision in patients aged 55–70 years [14].

### Subgroup analysis

When analysing all the secondary outcomes, the meta-analysis suggests that unexplained pain appears to be the most strongly associated adverse event, with the revision rate for unexplained pain increasing significantly in the obese population (Table 5). Unexplained pain also seems to account for the earliest revision rates in patients undergoing UKA with an average follow up time of 18 months until revision [1]. This may be due to the higher body mass causing bone overload. The revision of a UKA to TKA in the obese population appears to have been performed mostly in the first few years (average 2–2.2 years) in the studies included. This is in keeping with the literature where revision after UKA is seen in two peaks: early failure due to unexplained pain and late failure because of aseptic loosening [6, 13]. However, it must be stressed that due to the sparse number of patients and studies included in the subgroup analysis the results must be interpreted in context. Interestingly there appeared to be a statistically significant difference in the postoperative OKS (Table 5) between the two groups. However, this subgroup analysis only involved two studies and the mean difference may not be clinically important.

There were 757 patients included in the meta-analysis of infection. Ten patients had a superficial infection. Obesity was not associated with a significant increased risk of infection, odds ratio 1.73 (95% CI 0.47–6.31). This is unlike the outcomes of TKA where a meta-analysis of 20 studies has shown a significant increased risk of infection both in the short term and in the long term (OR 1.90, 95% CI 1.46–2.47) [21]. The risk of infection appears to be greater in TKA when compared with the infection rate in this meta-analysis of UKA (OR 1.73, 95% CI 0.47–6.31). Lum et al. looked at outcomes for 650 UKA and 1300 TKA in severely obese patients (BMI > 35) and found that there were lower deep infections rates and lower revision rates in those that underwent UKA [27]. In the recent two arm randomised controlled TOPKAT trial which compared UKA and TKA, there was no subgroup analysis comparing obese patients in either the TKA or the UKA group, however the revision rate in the UKA was lower than TKA [5].

It is generally accepted that obesity is associated with more intraoperative and postoperative complications [7, 19]. This may in part be due to the higher comorbidities in the obese population. Hence, it is interesting to note that only 4 studies documented postoperative complications and only one study documented intraoperative complications. No statistical model of the included studies adjusted for co-morbidities. It was difficult to assess the studies for baseline equivalence and confounding (Table 3) hence there is a clear need for future studies to adjust for the comorbidities using an appropriate comorbidity index tool in the statistical model.

### Prosthetic design

Prosthetic design plays an important influence on UKA performance and outcomes [2]. The study included a mixture of mobile bearing and fixed bearing UKA designs. There is evidence to suggest that fixed bearing UKA results in greater contact stress on the polyethylene inset which may eventually lead to a failure associated with wear or tibial component loosening [22]. Tibial component loosening is more frequent in the all-polyethylene designs than metal-backed prosthesis [22]. Although the increase in revision rate with obesity with the fixed bearing UKR (0.8% pa) was higher than with the mobile (0.2% pa) the difference was not statistically significant.

## Limitations

Owing to limited data and information available we were only able to explore the BMI thresholds of 30 and 35. The overall low number of studies heavily limited the resulting meta-analysis however by collating this available information, this study can be used as a reference point to inform knee surgeons when managing obese patients. Ideally each BMI category (e.g. 30–35, 35–40, 40–45 etc.) should be explored individually as some but not others might be contraindicated. Interestingly in the two studies that provided data about BMI > 45 there were no revisions. Setting a minimum follow-up period of one year, as part of the inclusion criteria would capture most of the early implant failures however one can argue that having no minimum follow up period would capture all the studies from a pragmatic analysis perspective. Another limitation of the study is to not include the change in patient reported outcome measures (PROMs) or postoperative PROMs as part of the meta-analysis however this was due to inconsistent reporting, missing data and heterogeneity.

The other main limitation of the meta-analysis is the decision to include all comparative studies, but this was made as it represented the best available evidence. Another challenge found was differentiating between studies that have been reported as prospective but are actually retrospective by design with prospective outcome data collection. However, the methodology scoring criteria applied showed that the studies were comparable and that pooling of the data was acceptable.

A key finding is the missing information presented in the studies. In particular, the lack of information on the performance and caseload of the surgeon performing the UKA. A study by Liddle et al. showed that surgeons performing fewer than 10 UKA’s per year had an 8-year survival of 87.9% compared with 92.4% for surgeons who performed 30 UKAs per year [26].

## Conclusions

Overall, this meta-analysis shows that a higher BMI does not lead to significantly worse outcomes of patients treated with a UKA. Therefore obese patients should not be excluded from undergoing a UKA based on BMI alone. However the revision rate for unexplained pain was the most strongly associated cause of revision in the obese population. There was a trend to increased revision rates with BMI > 30 and > 35 and with fixed bearing devices. Further studies are needed to increase the numbers in meta-analysis to explore activity levels, surgeon’s operative data, implant design and perioperative complications and revision in more depth. However, based on the available evidence obesity should not be considered a contraindication to UKA.

## Appendix

Search terms for online databases

Medline (Ovid) (*n* = 2224)

**Table:**

	Term	Studies
1	Obesity/	169,746
2	Body Mass Index/	118,030
3	Body weight/	184,387
4	Overweight/	22,474
5	Obes*.ti, ab	274,098
6	(Body mass index or body-mass index). ti, ab	166,193
7	bmi.ti, ab	129,016
8	Overweight.ti, ab	62,903
9	1 or 2 or 3 or 4 or 5 or 6 or 7 or 8	629,825
10	Arthroplasty, replacement, knee/	21,619
11	(Unicompartmental or uni-compartmental or UKA or UKR).ti, ab	2210
12	(knee adj3(arthroplast* or replace* or prosthes*)).ti,ab	30,537
13	Knee prosthesis/	11,096
14	10 or 12 or 13	35,781
15	11 and 14	1952

Web of Science (*n* = 2510)

Obesity OR overweight OR BMI OR “Body Mass Index” OR “Body-mass index” OR “Obes*” And “Knee arthroplast*” OR “Knee Prosth*” OR “Knee Replace*” OR “Unicompartmental Knee” OR UKA OR UKR OR “Uni-compartmental”.

PubMed Search (*n* = 3414).

(((((((((obesity) OR Body mass index) OR body weight) OR overweight[Title/Abstract]) OR obes*[Title/Abstract]) OR ((Body mass index[Title/Abstract] OR Body-mass index)[Title/Abstract]))))) AND ((((((((((prosthesis) OR arthroplasty) OR replacement) OR arthroplasty[tw]) OR replacement[tw]) AND knee)))).

## Abbreviations

BMI: Body Mass Index
CI: Confidence interval
KSS: Knee society score
LoE: Level of evidence
MD: Mean difference
MINORS: Methodological Index for non-randomised studies
NJR: National joint registry
OA: Osteoarthritis
OCEBM: Oxford Centre for Evidence-Based Medicine
OKS: Oxford Knee Score
OR: Odds ratio
PRISMA: Preferred reporting in systematic reviews and meta-analyses
PROMs: Patient reported outcome measures
TKA: Total knee arthroplasty
UKA: Unicompartmental knee arthroplasty
UKR: Unicompartmental knee replacement
WHO: World Health Organisation
